# Recombinant pediocin in *L* *actococcus lactis*: increased production by propeptide fusion and improved potency by co‐production with PedC

**DOI:** 10.1111/1751-7915.12285

**Published:** 2015-07-03

**Authors:** Alexandre Back, Frédéric Borges, Cécile Mangavel, Cédric Paris, Emmanuel Rondags, Romain Kapel, Arnaud Aymes, Hélène Rogniaux, Marija Pavlović, Auke J. van Heel, Oscar P. Kuipers, Anne‐Marie Revol‐Junelles, Catherine Cailliez‐Grimal

**Affiliations:** ^1^ Laboratoire d'Ingénierie des Biomolécules (LIBio)ENSAIAUniversité de Lorraine2 Avenue de la Forêt de HayeVandœuvre‐lès‐Nancy54518France; ^2^ Laboratoire Réactions et Génie des Procédés (LRGP)CNRS‐UMR 7274Université de Lorraine2 Avenue de la Forêt de HayeVandœuvre‐lès‐Nancy54518France; ^3^ INRA Unité Biopolymères Interactions Assemblages (UR1268)Rue de la GéraudièreNantes44316France; ^4^ Department of Molecular GeneticsGBB InstituteUniversity of GronningenNijenborgh 79747AGGroningenThe Netherlands

## Abstract

We describe the impact of two propeptides and PedC on the production yield and the potency of recombinant pediocins produced in *L* *actococcus lactis*. On the one hand, the sequences encoding the propeptides SD or LEISSTCDA were inserted between the sequence encoding the signal peptide of Usp45 and the structural gene of the mature pediocin PA‐1. On the other hand, the putative thiol‐disulfide oxidoreductase PedC was coexpressed with pediocin. The concentration of recombinant pediocins produced in supernatants was determined by enzyme‐linked immunosorbent assay. The potency of recombinant pediocins was investigated by measuring the minimal inhibitory concentration by agar well diffusion assay. The results show that propeptides SD or LEISSTCDA lead to an improved secretion of recombinant pediocins with apparently no effect on the antibacterial potency and that PedC increases the potency of recombinant pediocin. To our knowledge, this study reveals for the first time that pediocin tolerates fusions at the N‐terminal end. Furthermore, it reveals that only expressing the pediocin structural gene in a heterologous host is not sufficient to get an optimal potency and requires the accessory protein PedC. In addition, it can be speculated that PedC catalyses the correct formation of disulfide bonds in pediocin.

## Introduction

Bacteriocins from lactic acid bacteria are defined as antimicrobial proteinaceous compounds synthesized by ribosomes (Riley, 2009). They are used in food as bio‐preservatives and were lately suggested as drug candidates and probiotic promoting factors (Dobson *et al*., 2012). As bio‐preservatives, they can be added to food following three different strategies: *in situ* into fermented food by bacterial culture which constitutes the starter culture, directly in a purified or semi‐purified form (*e.g*. nisin A, nisaplin, Danisco) and as an ingredient based on a fermentation of a bacteriocin‐producing strain (pediocin PA‐1, ALTA 2431, Quest International) (Cotter *et al*., 2013). Utilization of bacteriocins as drugs in human or veterinary health has also been considered (Cotter *et al*., 2013; Van Heel *et al*., 2011). The industrial use of bacteriocins requires efficient downstream and upstream processes. However, low production rate by natural bacterial producers can dramatically impair further application of bacteriocins (Jack *et al*., 1996; Guyonnet *et al*., 2000; Jasniewski *et al*., 2008).

Heterologous expression is an efficient strategy to enhance bacteriocin production yields. The lactic acid bacterium *Lactococcus lactis* was successfully used to produce bacteriocins (Rodríguez *et al*., 2003) including pediocin PA‐1 (Horn *et al*., 1998; 1999; 2004; Reviriego *et al*., 2005, 2007a, 2007b; Martín *et al*., 2007a; Arqués *et al*., 2008), and could therefore be used as a host for large‐scale production. Besides, due to its generally recognized as safe status, *L. lactis* is an interesting bacterium for *in vivo* delivery of bacteriocins for probiotic purpose (Osmanagaoglu *et al*., 2010; Pontes *et al*., 2011; Dobson *et al*., 2012) or in fermented food (Renye and Somkuti, 2010). Another strategy, which aims at improving the secretion step and thereby the overall productivity, is to replace the wild‐type signal peptide of the recombinant protein by the Usp45 signal peptide (SP_usp45_), thus allowing secretion of pediocin PA‐1 through the secretory pathway (Li *et al*., 2011). However, although recombinant pediocin PA‐1 was successfully produced, little attention has been paid to the fact that the positive charges of pediocin PA‐1 might impair secretion through the secretory pathway. Indeed, the study of recombinant protein production in *L. lactis* showed that the insertion of the peptide LEISSTCDA between the signal peptide and the recombinant protein NucB allowed to increase production of NucB from 3 mg/L to 15 mg/L, highlighting the importance of the nature of the amino acids localized immediately after the cleavage site of the signal peptidase (Le Loir *et al*., 1998; Pontes *et al*., 2011). The class IIa bacteriocin divercin RV41 was successfully secreted through the secretory pathway with the remaining propeptide SD fused to the bacteriocin at the N‐terminus after the cleavage site. This propeptide SD results from the cloning procedure in the *Nsi*I restriction site that allows translational fusion between SP_usp45_ and the coding sequence of interest (Bermúdez‐Humarán *et al*., 2007). As previously suggested (Morello *et al*., 2008), it could be expected that the negative charge of aspartate in the propeptide SD might enhance protein secretion. However, this point, as well as the impact of the propeptide SD on the divercin RV41 specific activity, has not been investigated.

Pediocin PA‐1 is a model class IIa bacteriocin produced by several strains from *Pediococcus* and *Lactobacillus* species. This bacteriocin exhibits antibacterial activity against a wide spectrum of food‐borne pathogens and food spoilage gram‐positive bacteria (Rodríguez *et al*., 2002). As other class IIa bacteriocins, it affects both membrane permeability and membrane potential (Rodríguez *et al*., 2002). Mature pediocin PA‐1 is a 44 amino acid peptide containing four cysteine residues involved in two disulfide bonds (Henderson *et al*., 1992). The pediocin PA‐1 operon consists of the four genes named *pedA*, *pedB*, *pedC* and *pedD*, which encode the prepediocin PA‐1, the pediocin PA‐1 immunity protein, an accessory export protein and an ABC transporter (involved in the excision of the signal peptide and the secretion of pediocin) respectively (Venema *et al*., 1995).

The aim of this work was to investigate the efficiency of heterologous production of pediocin in *Lactococcus lactis* at the quantitative and the qualitative levels with a special focus on protein secretion and post‐translational modification.

## Results

### Impact of propeptides on secretion efficiency

**Figure 1:**
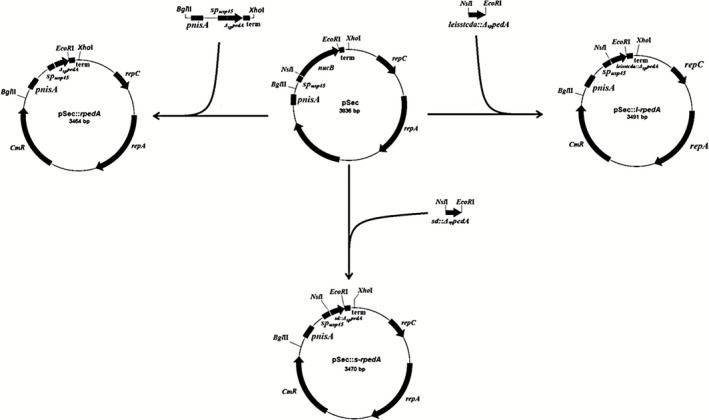
Construction of pSec derivatives. The restriction sites used for cloning purpose are indicated. The plasmid pSec and derivatives contain the nisin inducible promoter P*_nisA_*, the replication genes repA and repC, the gene conferring resistance to chloramphenicol and a transcriptional terminator term.

The three plasmids pSec::*rpedA*, pSec::*s‐rpedA* and pSec::*l‐rped*A were constructed with the aim to produce three different recombinant pediocins (Fig. 1). The three plasmids contain chimeras where *sp_usp45_*, which encodes the signal peptide of Usp45, was fused to the mature pediocin encoding sequence *Δ_sp_pedA*. In the plasmid pSec::*rpedA, sp_usp45_* was directly fused to *Δ_sp_pedA* which theoretically leads, after cleavage by the signal peptidase, to the release of a recombinant pediocin (Rpediocin) exhibiting the primary structure of the wild‐type mature pediocin PA‐1. In the plasmids pSec::*s‐rped* and pSec::*l‐rped*, the sequences encoding the propeptides SD and LEISSTCDA, respectively, were inserted between *sp_usp45_* and *Δ_sp_::pedA*. These plasmids theoretically lead to the secretion of recombinant fusion pediocins PA‐1, named S‐Rpediocin and L‐Rpediocin, that would exhibit the peptide SD and LEISSTCDA at the N‐terminus respectively. The three plasmids were used to transform *L. lactis*, and the resulting strains were cultivated in order to produce the expected recombinant pediocins. The strain *L. lactis* NZ9000_pSec::*nucB* was used as control. This control strain produces an extracellular staphylococcal nuclease (NucB) with no known antibacterial property.

For each producer, a dry weight of 0.7 g/L was measured, indicating that all strains approximately produced the same biomass. Furthermore, the maximum specific growth rate (0.8 h^−1^) was not significantly different between strains. The concentration of each recombinant pediocin was measured by Enzyme‐Linked Immunosorbent Assay (ELISA), and the specific production was determined by normalizing pediocin PA‐1 concentration to dry weight bacteria (Table 1). The strain *L. lactis* NZ9000_pSec::*rpedA* produced approximately 2.3 ± 0.4 μmol/g of recombinant pediocin, while the wild‐type producer strain *Lactobacillus plantarum* LMAX exhibited a specific productivity below 0.1 μmol/g. This result shows that specific production of recombinant pediocin by *L. lactis* is approximately 20 times more efficient than production of pediocin by the wild‐type strain. Subsequently, ELISA experiments revealed that the strains *L. lactis* NZ9000_pSec::*s‐rped* and *L. lactis* NZ9000_pSec::*l‐rped* produced approximately 3.5 ± 0.2 and 3.2 ± 0.3 μmol/g of pediocin respectively. The results thus show that the SD and LEISSTCDA propeptides allow a 1.5‐fold increase in secreted recombinant pediocin production.

**Table 1:** Specific production and antimicrobial activity

Strain	Specific production^a^^,^ b	MIC^a^^,^ c
*L. lactis* NZ9000_pSec::*rpedA*	2.3 ± 0.4	1.5 ± 0.3
*L. lactis* NZ9000_pSec::*s‐rpedA*	3.5 ± 0.2^d^	1.2 ± 0.1
*L. lactis* NZ9000_pSec::*l‐rpedA*	3.2 ± 0,3^d^	1.2 ± 0.2
*L. lactis* NZ9000*_*pSec::*s‐rpedA_*pOri23	4.0 ± 0,7	1.3 ± 0.5
*L. lactis* NZ9000_pSec::*s‐rpedA_*pOri*::pedC*	1.4 ± 0.1^e^	0.4 ± 0.1

a Mean ± standard deviation.

b Specific production was expressed as μmol pediocin PA‐1 equivalents/g of dry weight bacteria.

c MIC values of culture supernatants were expressed in μM pediocin PA‐1 equivalents.

d Indicate a significant difference (Student's *t*‐test, *P*‐value < 0,05) of specific production between the strains *L. lactis* NZ9000_pSec::*s‐rpedA L. lactis* NZ9000_pSec::*l‐rpedA* and the control strain *L. lactis* NZ9000_pSec::*rpedA*.

e Indicate a significant difference (Student's *t*‐test, *P*‐value < 0,05) of specific production between the strains *L. lactis* NZ9000*_*pSec*::s‐rpedA_*pOri::*pedC* and the control strain *L. lactis* NZ9000_pSec::*s‐rpedA_*pOri::*pedC*.

### Antibacterial activity

**Figure 2:**
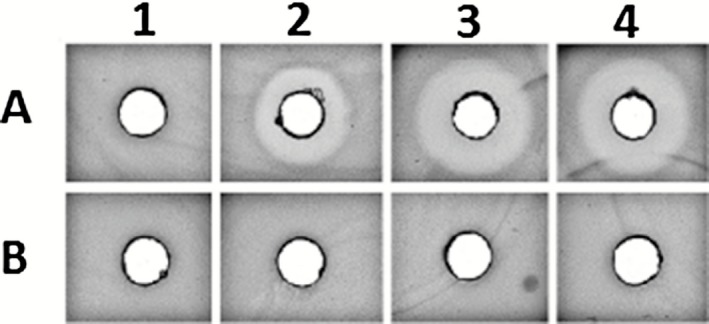
Antibacterial activity of recombinant pediocins. The antibacterial activity of recombinant pediocins was assessed on agar plate using the sensitive strain *C* *. maltaromaticum* DSM20730_pSec::nucB_pOri23 (line A) or the pediocin PA‐1 resistant strain *C* *. maltaromaticum* DSM20730_pSec::nucB_pOri::pedB (line B) as target strains. Pictures in columns 1, 2, 3, 4 show the inhibitory activity of the culture supernatants of the negative control (heterologous production of NucB from *S* *taphylococcus aureus*), Rpediocin (recombinant mature pediocin PA‐1), S‐Rpediocin (recombinant pediocins PA‐1 fused to the peptide SD at the N‐terminus) and L‐Rpediocin (recombinant pediocins PA‐1 fused to the peptide LEISSTCDA at the N‐terminus) respectively.

The antibacterial activity of supernatants was assessed by agar‐well diffusion assay using *Carnobacterium maltaromaticum* DSM20730_pSec::*nucB*_pOri23 as target strain. Inhibition areas were obtained from the culture supernatants of the three strains *L. lactis* NZ9000_pSec::*rpedA*, *L. lactis* NZ9000_pSec::*s‐rpedA* and *L. lactis* NZ9000_pSec::*l‐rpedA* (Fig. 2A, panels 2, 3 and 4). No inhibition was obtained with the culture supernatant of the control strain *L. lactis* NZ9000_ pSec::*nucB* (Fig. 2A, panel 1). Moreover, no inhibition was observed when the same experiment was carried out with an indicator strain previously transformed with pOri::*pedB*, which confers immunity to pediocin PA‐1 (Fig. 2B, panels 2, 3 and 4). These results show the three strains *L. lactis* NZ9000_pSec::*s‐rpedA*, *L. lactis* NZ9000_pSec::*l‐rpedA* and *L. lactis* NZ9000_pSec::*rpedA* produce active recombinant pediocins. The supernatants containing the recombinant pediocins Rpediocin, S‐Rpediocin and L‐Rpediocin exhibited minimal inhibitory concentration (MIC) values of 1.5 ± 0.3 μM, 1.2 ± 0.1 μM and 1.2 ± 0.2 μM (Table 1) respectively. These results show the propeptides SD and LEISSTCDA have no major impact on the MIC value of the supernatants containing recombinant pediocins.

### Co‐production of PedC with S‐Rpediocin

**Figure 3:**
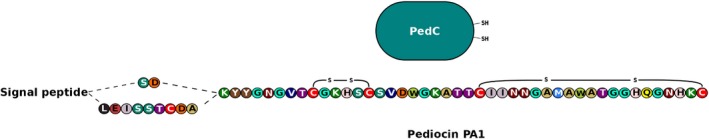
Strategies used to quantitatively and qualitatively act on pediocin production in *L* *lactis*. Two propeptides were tested to improve secretion, and PedC was coexpressed with recombinant pediocin to improve its potency.

The potency of the recombinant pediocins produced in *L. lactis* was compared with the wild‐type pediocin produced by *Pediococcus acidilactici*. Surprisingly, the wild‐type pediocin PA‐1 produced from *P. acidilactici* exhibited a MIC value of 0.05 μM, and was thus approximately 30 times more active than the recombinant pediocins. This result suggests that the structure of recombinant pediocins is different from the wild‐type pediocin (Fig. 3). Although class IIa bacteriocins are described to be non‐post‐translationally modified, they all exhibit at least one disulfide bond. It was previously described that disulfide bonds are important for the antibacterial activity of pediocin PA‐1 (Fimland *et al*., 2000). Even if disulfide bond formation can be spontaneous, this is a slow and aspecific process that can require enzymatic catalysis in bacteria (Kadokura *et al*., 2003). It was hypothesized that such enzymes could allow correct formation of disulfide bonds in wild‐type pediocin PA‐1 bacterial producers (Fimland *et al*., 2000). Lately, it was shown that thiol‐disulfide oxidoreductases (TDORs), which belong to the thioredoxin superfamily and are characterized by a conserved CXXC motif, are involved in the post‐translational maturation of the bacteriocins sublancin 168 and BlpG_st_ (Dorenbos *et al*., 2002; Fontaine and Hols, 2008). Interestingly, sequence analysis of the pediocin PA‐1 operon *pedABCD* revealed that *pedC* encodes a putative secreted protein with a CXXC motif and a thioredoxin‐like fold. It can be therefore hypothesized that PedC is a TDOR and allows native disulfide bond formation of pediocin PA‐1.

**Figure 4:**
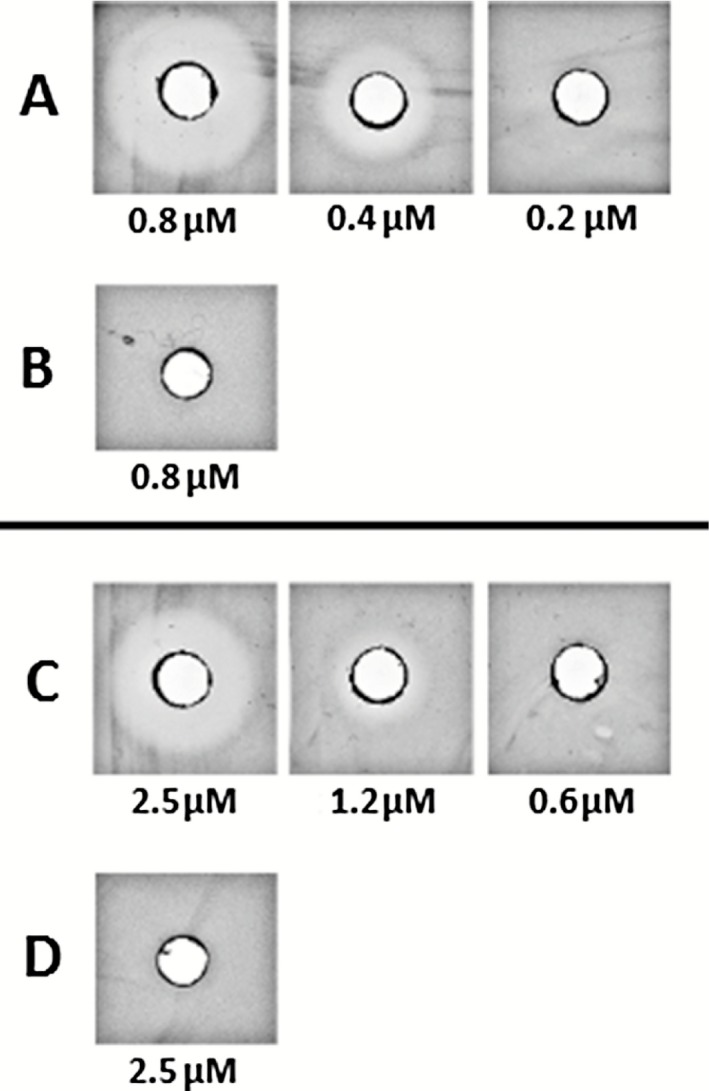
The antimicrobial activity of recombinant pediocin produced by *L* *. lactis* NZ9000_pSec::*s‐rped*_pOri::pedC (line A and line B) and *L* *. lactis* NZ9000_pSec::*s‐rped*_pOri23 (line C and line D) was assessed on agar plate using the sensitive strain *C* *. maltaromaticum* DSM20730_pSec::nucB_pOri23 (line A and C) or the pediocin PA‐1 resistant strain *C* *. maltaromaticum* DSM20730_pSec::nucB_pOri::pedB as target strains (line B and D). Picture in lines A and C show serial twofold dilutions of culture supernatants of strains *L* *. lactis* NZ9000_pSec::*s‐* rpedA_pOri::pedC and *L* *. lactis* NZ9000_pSec::*s‐* rpedA_pOri23 respectively. The concentrations below the pictures are expressed in μM equivalents of pediocin PA‐1.

The encoding sequence of *pedC* was cloned into pOri23, and the resulting plasmid was used to transform *L. lactis* NZ9000_pSec::*s‐rpedA*. In parallel, the same recipient strain was transformed with pOri23, and the resulting strain was used as a control strain. Inhibition assays resulted in broader growth inhibition halos for the strain transformed with pOri::*pedC* (Fig. 4A) than for the strain transformed with pOri23 (Fig. 4C). Consistently, the MIC value of the recombinant pediocin decreased from 1.2 ± 0.1 μM to 0.4 ± 0.1 μM when *pedC* was co‐expressed with the recombinant pediocin (Table 1). Besides, the strain producing PedC exhibited a three times lower specific productivity compared with the control strain (Table 1). These results show that even if lower amounts of recombinant pediocin are produced, a higher activity was obtained with strain co‐producing the recombinant pediocin and PedC. When the same experiment was carried out with the indicator strain transformed with the pOri::*pedB* plasmid, which encodes the pediocin PA‐1 immunity factor, no inhibition was observed (Fig. 2B and D). Furthermore, no inhibition was observed when the control strain *L. lactis* NZ9000_pSec::*nucB* was transformed with pOri::*pedC*, indicating that PedC does not exhibit detectable antibacterial activity in our experimental conditions (data not shown). These results show that PedC allows increasing the antibacterial potency of the recombinant pediocin S‐Rpediocin in *L. lactis*.

### PedC homologues as putative antibacterial activity promoting factors of bacteriocins

**Figure 5:**
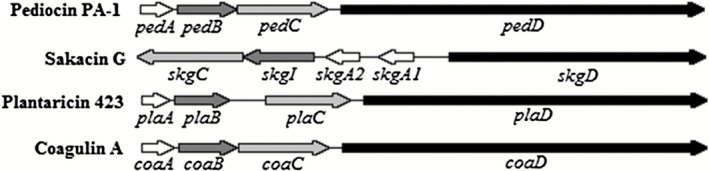
Genetic structure of gene clusters of class IIa bacteriocin containing a pedC homologue.

Previous studies already showed that the TDORs BlpG_st_ and BdbA are involved in disulfide bond formation of the bacteriocin thermophilin 13 (Fontaine and Hols, 2008) and the bacteriocin sublancin 168 (Dorenbos *et al*., 2002) respectively. BLASTP analysis revealed that BlpG_st_ shares 26% of identity (e‐value = 2,00E‐008) with PedC, and that BdbA shares 17% of identity (e‐value = 4,00E‐008) with PedC. Furthermore, BlpG_st_ and PedC both exhibit a thioredoxin‐like fold (Interproscan accession number IPR012336), a CXXC motif and a bacteriocin transport accessory protein‐like fold (Interproscan accession number IPR005985). The proteins SkgC, PlaC, CoaC which are transport accessory proteins of the class IIa bacteriocins sakacin G, plantaricin 423 and coagulin A (Fig. 5), respectively, appears to have the same thioredoxin‐like fold and the same bacteriocin transport accessory protein‐like fold as PedC. Moreover, PedC shares 47% (e‐value = 1,00E‐056), 99% (e‐value = 1,00E‐132) and 99% (e‐value = 3,00E‐132) of identity with SkgC, PlaC and CoaC respectively. This suggests that SkgC, PlaC and CoaC might enhance the activity of the class IIa bacteriocins sakacin G, plantaricin 423 and coagulin A respectively.

## Discussion

The general secretion (Sec) system of *L. lactis* was widely used to improve secretion of recombinant proteins including bacteriocins (Herranz and Driessen, 2005; Bermúdez‐Humarán *et al*., 2007; Borrero *et al*., 2011a, 2011b; Jiménez *et al*., 2013) such as the bacteriocin pediocin PA‐1 (Martín *et al*., 2007a; Li *et al*., 2011). In order to allow secretion via the Sec system in *L. lactis*, the signal peptide of the Usp45 protein SP_usp45_ is commonly fused to the N‐terminus of recombinant proteins (Morello *et al*., 2008). Since it is thought that positive charges impair secretion through the secretory pathway, sequences encoding SD or LEISSTCDA propeptides were fused to the nucleotide sequence encoding the mature pediocin PA‐1 (*Δ_sp_pedA*) at the 5′ end. Both propeptides allowed the improvement of the production of recombinant pediocins with no marked difference between propeptides SD and LEISSTCDA.

The effect of the signal peptide SP_usp45_ and the propeptide LEISSTCDA were previously described to improve the overall productivity in *L. lactis* (Le Loir *et al*., 1998; 2001; Langella and Le Loir, 1999; Freitas *et al*., 2005; Nouaille *et al*., 2005; Zhang *et al*., 2010). The replacement of the signal peptide of NucB by the signal peptide of the major secreted protein Usp45 (SP_usp45_) increased NucB secretion. It was hypothesized that enhancement of protein secretion resulted from a more efficient recognition of the SP_usp45_ by the secretory secretion machinery of *L. lactis*. Moreover, when the propeptide LEISSTCDA was inserted between SP_usp45_ and NucB, secretion was further improved. The underlying mechanism is still unknown, it is speculated that the negative charges of the propeptide LEISSTCDA would increase the cleavage efficiency by signal peptidase and/or would fold into a conformational state that would lead to optimal secretion efficiency. This optimal secretion efficiency would therefore allow the protein precursor to be less sensitive to intracellular degradation or would help to escape intracellular degradation thus increasing the production yield (Le Loir *et al*., 2005). A similar mechanism could explain the higher productivity of the recombinant pediocins fused to SD or LEISTCDA propeptides.

Propeptides SD and LEISSTCDA did not have any significant impact on the antibacterial specific activity of recombinant pediocin PA‐1. Thorough analysis of the impact of additional amino acid residues on specific activity of sakacin P revealed that an extra lysine residue at the C‐terminal end had no marked effect on antibacterial activity. However, a lysine residue added at the N‐terminus strongly reduced the potency of sakacin P (Kazazic *et al*., 2002). These results indicate that adding amino acid residues at the N‐terminal end of class IIa bacteriocins can have various impacts on their potency and peptide engineering implying peptide fusion at the N‐terminus would require checking the impact on antibacterial potency of pediocin‐like bacteriocins.

It was shown that all recombinant pediocins in the culture supernatants, including Rpediocin, are less active than the wild‐type pediocin PA‐1 produced by *P. acidilactici*. Similar results were already reported for pediocin PA‐1, enterocin A, divercin RV41 and sakacin A, and it was hypothesized that recombinant bacteriocins might be subjected to self‐aggregation, degradation or could be misfolded (Bermúdez‐Humarán *et al*., 2007; Martín *et al*., 2007a, 2007b; Borrero *et al*., 2011b; Jiménez *et al*., 2013). Another hypothesis would be linked to the fact that pediocin PA‐1 has two disulfide bonds. The disulfide bond at the N‐terminus is conserved in class IIa bacteriocins, whereas the disulfide bond at the C‐terminus is less conserved and is important for both pediocin PA‐1 spectrum and stabilization inside the bacterial membrane (Fimland *et al*., 1996; 2000; 2005). When heterologous production of pediocin PA‐1 is performed using *Lactobacillus sakei*, non‐native disulfide bonds are formed (Fimland *et al*., 2000). We hypothesized that similar cystein mispairing were formed in recombinant pediocin PA‐1 produced in *L. lactis*. Sequence analysis of the *pedABCD* operon revealed *pedC* would encode a protein with a putative thioredoxin fold and a CXXC catalytic site, suggesting a probable role in the status of the pediocin PA‐1 disulfide bonds. In this study, co‐production of PedC and the recombinant pediocin S‐Rpediocin led to the production of a bacteriocin with higher potency. Although PedC is described as an accessory transport protein, it was indeed concluded to be essential for secretion by the dedicated secretion system in both *Pediococci* and *Escherichia coli* (Venema *et al*., 1995). Nevertheless, since the amount of pediocin PA‐1 produced was not measured, it remains unclear whether the absence of activity recorded in this study was due to a decrease of secretion efficiency and/or of potency. In line with this, another study demonstrated that PedD was sufficient to allow secretion of pediocin PA‐1 in *E. coli* (Bukhtiyarova *et al*., 1994). Our results further support that PedC increases the potency of pediocin PA‐1. Sequence analyses showed that *pedC* homologous genes are genetically linked to genes encoding the class IIa bacteriocin sakacin G, plantaricin 423 and coagulin A suggesting that TDORs are critical functions for these bacteriocins. Two other studies revealed the role of TDORs in the potency of bacteriocins belonging to other classes: the class VI sublancin 168 (Dorenbos *et al*., 2002) and the class IIb thermophilin 9 (Fontaine and Hols, 2008), suggesting that the role of TDORs in the potency of bacteriocins would be not restricted to one particular class of bacteriocins.

When PedC was co‐expressed with recombinant pediocin S‐Rpediocin, a lower concentration of S‐Rpediocin was obtained in the culture supernatant. Sequence analysis of PedC suggested it contains a secretory‐dependent signal peptide. In addition, *pedC* is under the control of the strong constitutive promoter p23 (Van der Vossen *et al*., 1987). Therefore, it can be speculated that PedC competes with the recombinant pediocin for the recruitment by the secretory machinery for secretion.

This study revealed that the model class IIa bacteriocin pediocin PA‐1 is sufficiently flexible to tolerate fusions at the N‐terminal end. This property was used here to improve secretion efficiency and could also be used for other purposes such as genetic engineering of innovative antibacterial compounds as previously shown for other bacteriocins (Qiu *et al*., 2003; Acuña *et al*., 2012). Moreover, this work revealed that solely expressing the structural gene of class IIa pediocin PA‐1 in a heterologous host may not be sufficient to get an optimal antibacterial activity, and that PedC might be helpful to enhance the activity of recombinant bacteriocins, probably by increasing the concentration of correct disulfide bond containing peptides. Furthermore, these results suggest that the protein PedC is responsible for the formation of disulfide bonds in pediocin PA‐1 in the wild‐type context.

## Experimental procedures

### Bacterial strains, culture conditions and plasmids

Bacterial strains and plasmids used in this study are listed in Table 2. *Lactococcus lactis* NZ9000 was grown at 30°C in M17 (Difco, Sparks, USA) supplemented with 1% (w/v) glucose (GM17 medium). *Carnobacterium maltaromaticum* DSM20730 was grown in Trypton Salt Broth medium (TSB; Biomérieux, Marcy‐l'Étoile, France) supplemented with 6 g/L yeast extract (TSBYE) at 30°C. *Lactobacillus plantarum* LMAX, which was isolated from HOLDBAC *Listeria* (Danisco), was grown in Man, Rogosa and Sharpes medium (MRS, Biokar, Beauvais, France) at 30°C. *Lactococcus lactis* NZ9000 transformants were grown in GM17 containing 10 μg/ml chloramphenicol or/and 10 μg/ml erythromycin (Sigma‐Aldrich, Saint‐Louis, USA). *Carnobacterium maltaromaticum* DSM20730 transformants were grown in TSBYE containing 5 μg/ml chloramphenicol or/and 5 μg/ml of erythromycin.

**Table 2:** Strains, plasmids and synthetic genes used in this study

Plasmid, strain and synthetic genes	Description	Reference
**Strains**		
*L. lactis* NZ9000	MG1363 derivative; *pepN::nisRK+*	(Kuipers *et al*., 1998)
*C. maltaromaticum* DSM20730^a^	Pediocin sensitive strain	(Hiu *et al*., 1984)
*Lb plantarum* LMAX	Natural pediocin producer, isolated from HOLDBAC *Listeria*	Danisco
*L. lactis* NZ9000_pSec::*nucB*	Carry the plasmid pSec which contains the *nucB* gene under the control of P*_nisA_*	This study
*L. lactis* NZ9000_pOri::*pedB*	Carry the plasmid pOri::*pedB* which contains the *pedB* gene under the control of P23	This study
*L. lactis* NZ9000_pSec::*rpedA*	Carry the plasmid pSec::*rpedA* which contains the *Δ_sp_pedA* gene under the control of P*_nisA_*.	This study
*L. lactis* NZ9000_pSec::*s‐rpedA*	Carry the plasmid pSec::*s‐rpedA* which contains the *sd::Δ_sp_pedA* gene under the control of P*_nisA_*	This study
*L. lactis* NZ9000_pSec::*l‐rpedA*	Carry the plasmid pSec::*l‐rpedA* which contains the *leisstcda::Δ_sp_pedA* gene under the control of P*_nisA_*	This study
*L. lactis* NZ9000_pSec_pOri::*pedC*	Carry the plasmid pSec which contains the *nucB* gene under the control of P*_nisA_* and pOri::*pedC* which contains the *pedC* gene under the control of P23.	This study
*L. lactis* NZ9000_pSec::*s‐rpedA_*pOri23	Carry the plasmid pSec::*s‐rpedA* which contains the *sd::Δ_sp_pedA* under the control of P*_nisA_* and the plasmid pOri23	
*L. lactis* NZ9000_pSec::*s‐rpedA_pOri::pedC*	Carry the plasmid pSec::*s‐rpedA* which contains the *sd::Δ_sp_pedA* under the control of P*_nisA_* and pOri::*pedC* which contains the *pedC* gene under the control of P23.	This study
*C. maltaromaticum* DSM20730_pSec::*nucB*_pOri23	Pediocin sensitive strain resistant to erythromycin and chloramphenicol	This study
**Plasmids**		This study
pSec::*nucB*	CmR, P*_nisA_*, *sp_usp45_*, *nucB*, *repA*, *repC*	Bermúdez‐Humarán *et al*., 2007)
pOri23	ErmR, p23, *repD*, *repE*	Que *et al*., 2000)
pOri::*pedB*	ErmR, p23, pOri23 derivative carrying *pedB*	This study
pSec::*rpedA*	CmR, pSec derivative carrying *sp_usp45_::rpedA* and encoding secreted Rpediocin	This study
pSec::*s‐rpedA*	CmR, pSec: derivative carrying *sp_usp45_::s‐rpedA* and encoding secreted S‐Rpediocin	This study
pSec::*l‐rpedA*	CmR, pSec derivative carrying *sp_usp45_::l‐rpedA* and encoding secreted L‐Rpediocin	This study
pOri::*pedC*	ErmR, p23, pOri23 derivative carrying *pedC*	This study
**Synthetic genes**	**Amino acid sequence**	
*sd::Δ_sp_pedA*	b **tctgata**aatattatggtaatggagttacttgtggaaaacattcatgttctgttgattggggtaaagctacaacttgtattattaataatggagctatggcatgggctactggtggacatcaaggtaatcataaatgttaa	Genscript
*leisstcda::Δ_sp_pedA*	b **ttagaaatttcatcaacatgtgatgct**aatattatggtaatggagttacttgtggaaaacattcatgttctgttgattggggtaaagctacaacttgtattattaataatggagctatggcatgggctactggtggacatcaaggtaatcataaatgttaa	Genscript

a The strain was from *Deutsche Sammlung von Mikroorganismen und Zellkulturen* collection.

b The nucleotide sequences encoding propeptides SD and LEISSTCDA fused to the N‐terminus of pediocin PA‐1 are in bold.

### Electroporation

Electrocompetent *L. lactis* NZ9000 was obtained as previously described (Holo and Nes, 1989). For electrocompetent *C. maltaromaticum*, an overnight culture was diluted 10‐fold in fresh TSBYE medium and incubated at 30°C until an OD_600__nm_ of 0.7 was reached. Then bacteria were harvested by centrifugation at 6000 g for 15 min. Following three washes in ice‐cold 10% glycerol solution, the pellet was re‐suspended in 1 ml of the same washing solution and stored at –80°C. The electric pulse was delivered with a Gene‐Pulser (Bio‐Rad Laboratories, Richmond, USA.) set up at 25 μF, 2.1 kV and 200 Ω. Then, the suspension was mixed with 4 ml of TSBYE and incubated 4 h at 30°C prior plating on selective medium containing 5 μg/ml chloramphenicol or/and 5 μg/ml erythromycin.

### Plasmid construction

All the polymerase chain reaction (PCR) products used for cloning purpose were obtained with the oligonucleotide primers (Eurogentec, Seraing, Belgique) described in Table 3 by using the high‐fidelity thermostable polymerase *Pfx*50 (Invitrogen, Carlsbad, USA). The expression vectors pSec::*s‐rpedA* and pSec::*l‐rpedA* were constructed by using pSec::*nucB* as recipient vector. Polymerase chain reaction product PCRSD and PCRLEI (Table 2) were digested with *Nsi*I and *Eco*RI (New England Biolabs, Massachusetts, Ipswich, USA) and were ligated into the pSec expression vector previously digested with the same enzymes to generate pSec::*s‐rpedA* and pSec::*l‐rpedA* expression vectors respectively. The plasmid pSec::*rpedA* was constructed by deleting the SD encoding sequence by crossover PCR. To do so, the PCR products named PEDA1 and PEDA2 which flank the SD encoding region, were obtained by using pSec::*s‐rpedA* as DNA matrix. The product named PEDA3 was obtained by crossover PCR by using PEDA1 and PEDA2 as DNA matrix and was subsequently digested with *Bgl*II and *Xho*I and cloned into the pSec expression vector previously digested with the same enzymes in order to generate the expression vector pSec::*rpedA*. Plasmid pOri::*pedC* was obtained by digesting the PCR product PEDC with *Bam*HI and *Pst*I (FastDigest, Thermo Fisher Scientific, Waltham, Massachusetts, USA). The plasmid pOri::*pedB* was constructed similarly by using the primers described in Table 3. All the recombinant plasmids were obtained by using the ligation mixes to electrotransform *L. lactis* NZ9000. The integrity of plasmid inserts was checked by DNA sequencing according to the method of Sanger (GATC‐BIOTECH, Konstanz, Germany).

**Table 3:** Primers and PCR products

PCR product name	Matrix	Primers^a^
PEDB	*Lb. plantarum* LMAX	Forward : ATCG**GGATCC**TAA*AAAGGGAG*GCCAAATATAATGAATAAGACTAAGTCGGAA
		Reverse : ATCG**CTGCAG**CTATTGGCTAGGCCACGT
PCRSD	*sd::Δ_sp_pedA*	Forward : CCCCCCCC**ATGCAT**CTGATAAATATTATGGTAATGGAGTTACTTGTGGA
		Reverse : CCCCCC**GAATTC**ACTAGTCCTTAACATTTATGATTACCTTG
PCRLEI	*leisstcda::Δ_sp_pedA*	Forward : CCCCCCCC**ATGCAT**TAGAAATTTCATCAACATGTGATGCTAAATAT
		Reverse : CCCCCC**GAATTC**ACTAGTCCTTAACATTTATGATTACCTTG
PEDA1	pSec::*s‐rpedA*	Forward : ACAGCTCCAAGATCTAGTCTT
		Reverse : AGTAACTCCATTACCATAATATTTTGCATAAACACCTGACAACGG
PEDA2	pSec::*s‐rpedA*	Forward : CCGTTGTCAGGTGTTTATGCAAAATATTATGGTAATGGAGTTACT
		Reverse : ACATGCTGAAGAGCATCTCATT
PEDA3	PEDA1 and PEDA2	Forward : ACAGCTCCAAGATCTAGTCTT
		Reverse : ACATGCTGAAGAGCATCTCATT
PEDC	*Lb. plantarum* LMAX	Forward : GGGGGG**GGATCC**TAA*AAAGGGAG*GCCAAATATAATGTCTAAGAAATTTTGGTCAAA
		Reverse : GGGGGG**CTGCAG**CTACTGATTATTGTAATCAGC

a Restriction sites are in bold, Ribosome Binding Site sequences are italicized.

### Heterologous expression

For strains containing pSec::*nucB* or pSec derivatives, a culture incubated for 17 h was used to inoculate 200 ml of GM17 supplemented with chloramphenicol (10 μg/ml) in a 250 ml flask at an initial OD_600__nm_ of 0.02. Cultures were grown at 100 r.p.m. stirring (Amplitude: 20 mm; Sanyo, Osaka, Japan) and at 30°C. For strains containing both pSec::*nucB* or derivatives, and pOri23 or derivatives, a culture incubated for 17 h was used to inoculate 200 ml of GM17 medium supplemented with chloramphenicol (2 μg/ml) and erythromicyn (2 μg/ml) in a 250 ml flask at an initial OD_600__nm_ of 0.1. For all strains, heterologous production was induced with 100 ng/ml of semi‐purified nisin (balance sodium chloride and denatured milk solids containing 2.5% of nisin from Sigma‐Aldrich, St Louis, USA) when OD_600__nm_ reached 0.4–0.5. After 24 h of incubation, cell dry weights were determined gravimetrically. Then the supernatants were collected after centrifugation (5000 g; 15 min). Each supernatant was neutralized with 6M NaOH, sterilized by microfiltration (0.2 μm) and stored at −20°C. The experiments were independently performed three times.

### ELISA

Recombinant pediocin concentrations were measured by ELISA. Rabbit serum containing anti‐pediocin PA‐1 polyclonal antibodies was purchased from Eurogentec (Seraing, Belgique). Goat anti‐rabbit IgG antibodies coupled with Horseradish peroxidase (HRP) were purchased from Sigma‐Aldrich (St Louis, USA). Between each step described below, the microtitre plate was washed eight times with washing buffer (phosphate buffer saline containing 0.05% of Tween 20). Microtitre plate wells (Corning, New york, USA) were coated overnight at 4°C with 200 μl of filtered culture supernatant containing recombinant pediocins previously diluted with phosphate buffer saline (PBS) when required. Wells were subsequently blocked with 300 μl of skimmed milk (10% w/v in PBS, Régilait, Saint‐Martin‐Belle‐Roche, France) for 1 h at room temperature. Wells were then incubated with 150 μl of anti‐pediocin polyclonal antibodies (1:1000 in PBS) for 1 h at room temperature, followed by incubation with 100 μl of HRP‐labelled goat anti‐rabbit IgG (1:2000 in PBS) for 1 h. Bound secondary antibodies were detected by adding 100 μl of 3,3′,5,5′‐tetramethylbenzidine (TMB, Thermo Fisher Scientific, Waltham, USA). The reaction was stopped with 100 μl of 2M H_2_SO_4_ and absorbance was read at 450 nm. Concentration of recombinant pediocins in the supernatants were extrapolated from a calibration curve constructed using purified pediocin PA‐1 from *P. acidilactici* (Sigma‐Aldrich, St Louis, USA) diluted with PBS and were expressed in μM equivalents of pediocin PA‐1.

### Antimicrobial assay

Minimal inhibitory concentration of pediocin PA‐1 and recombinant pediocins were determined by well diffusion assay on agar plates (Mathieu *et al*., 1993) by using the strain *C. maltaromaticum* DSM20730_pSec_pOri23 as indicator. Minimal inhibitory concentration is defined as the lowest concentration exhibiting a distinct inhibition zone after 24 h at 30°C.

### Sequence analysis

The nucleotide sequence of putative thiol disulfide oxidoreductases were analysed using blast (Altschul *et al*., 1997), SignalP (Nielsen *et al*., 1997), Conserved Domain Database CDD (Marchler‐Bauer and Bryant, 2004; Marchler‐Bauer *et al*., 2007; 2013) and Interproscan (Quevillon *et al*., 2005). The amino acid sequence of PedC (accession number: P37249), BdbA (accession number: P68569), SkgC (accession number: B2LS01 ), CoaC (accession number: Q9EZB1), PlaC (accession number: Q93FV5), BlpG_st_ (accession number: Q03J39) used for analysis are from UniProt.

## Conflict of interest

The authors declare no conflict of interests.
